# Single-cell genetic analysis validates cytopathological identification of circulating cancer cells in patients with clear cell renal cell carcinoma

**DOI:** 10.18632/oncotarget.25102

**Published:** 2018-04-13

**Authors:** Lucile Broncy, Basma Ben Njima, Arnaud Méjean, Christophe Béroud, Khaled Ben Romdhane, Marius Ilie, Veronique Hofman, Jane Muret, Paul Hofman, Habiba Chaabouni Bouhamed, and Patrizia Paterlini-Bréchot

**Affiliations:** ^1^ INSERM Unit 1151, Faculté de Médecine Paris Descartes, Paris, France; ^2^ Genetics and Pathology Departments, University of Tunis, Tunis, Tunisia; ^3^ Service d’Urologie, Hôpital Européen Georges Pompidou, Paris, France; ^4^ Aix Marseille University, INSERM, MMG, Marseille, France; ^5^ APHM, Hôpital TIMONE Enfants, Laboratoire de Génétique Moléculaire, Marseille, France; ^6^ Laboratoire de pathologie clinique et Biobank BB-0033-00025, Centre Hospitalo-Universitaire de Nice, Nice, France; ^7^ Institut Curie, PSL Research University, Département d’Anesthésie Réanimation Douleur, Paris, France; ^8^ Laboratoire de Biochimie A, Hôpital Necker-Enfants Malades, Paris, France

**Keywords:** liquid biopsy, circulating cancer cells, ISET^®^ (Isolation by Size of Tumour/Trophoblastic Cells) technology, clear cell renal cell carcinoma, VHL mutation

## Abstract

**Context:**

Circulating Rare Cells (CRC) are non-haematological cells circulating in blood. They include Circulating Cancer Cells (CCC) and cells with uncertain malignant features (CRC-UMF) according to cytomorphology. Clear cell renal cell carcinomas frequently bear a mutated Von Hippel-Lindau (VHL) gene.

**Aim:**

To match blind genetic analysis of CRC and tumor samples with CRC cytopathological diagnosis.

**Results:**

29/30 patients harboured CRC (20 harboured CCC, 29 CRC-UMF) and 25/29 patients carried VHL mutations in their tumour. 205 single CRC (64 CCC, 141 CRC-UMF) provided genetic data. 57/57 CCC and 104/125 CRC-UMF from the 25 patients with VHL-mutated tumor carried the same VHL mutation detected in the tumor. Seven CCC and 16 CRC-UMF did not carry VHL mutations but were found in patients with wild-type VHL tumor tissue.

**Conclusions:**

All the CCC and 83,2% (104/125) of the CRC-UMF were found to carry the same VHL mutation identified in the corresponding tumorous tissue, validating cytopathological identification of CCC in patients with clear cell renal cell carcinoma.

**Methods:**

The blood of 30 patients with clear cell renal cell carcinoma was treated by ISET^®^ for CRC isolation, cytopathology and single-cell VHL mutations analysis, performed blindly and compared to VHL mutations of corresponding tumor tissues and leukocytes.

## INTRODUCTION

Circulating Rare Cells (CRC) are rare and heterogeneous cells circulating in blood and deriving from organs [1]. They include circulating tumors cells (CTC) [2] as well as non-tumorous, non-haematological cells, mainly of epithelial or endothelial origin, to be distinguished from cancer cells.

CTC are thought to represent an accessible source of tumor material for monitoring tumor invasion and response to treatment, and for detecting predictive molecular biomarkers to identify patients eligible for targeted treatments [3].

CTC can be isolated either by marker-dependent or -independent technologies. Importantly though, because CTC populations consist of very heterogeneous phenotypes and may express epithelial or mesenchymal markers or sometimes both [4–6], markers alone are not diagnostic and cytopathology remains the reference method for circulating cancer cells diagnosis [1]. Furthermore, marker-dependent isolation approaches may lead to selection biases, false positive and false negative results [1, 7, 8]. Isolation by SizE of Tumor/Trophoblastic cells (ISET^®^) is a particularly sensitive marker-independent technology that relies on the fact that blood cells are the smallest cells in the body and takes advantage from the larger size of CRC, including all types of cancer cells derived from solid cancers. CRC are retained on a filter, while erythrocytes are lysed and the majority of leucocytes are lost through the 8 micron pores [9]. This permits a very sensitive isolation of CRC from blood, without marker-related bias, keeping them intact, thereby allowing their cytopathological diagnosis, and further immunomorphological and molecular analysis. The superior sensitivity of ISET^®^ has been demonstrated by independent studies both *in vitro* [5, 10–12] and *in vivo* [13], including in comparative tests (reviewed in [14]).

In this setting, since the term circulating tumor cells (CTC) has been applied to cells extracted from blood using epithelial markers and is therefore associated to possible false positive and false negative results, the term circulating cancer cell (CCC) has been introduced to strictly designate cancer cells, of epithelial or mesenchymal origin, isolated from blood without bias and diagnosed by cytopathology [1].

Under cytopathological analysis, CRC can be distinguished as CRC with malignant features (CRC-MF), also called Circulating Cancer Cells (CCC) and CRC with uncertain malignant features (CRC-UMF). Importantly, CRC isolated by ISET^®^ can undergo further characterization such as genetic analyses at single-cell level [9, 14–18] which could help the cytopathological diagnosis in difficult cases provided that the tumor displays tumor-specific genetic mutations.

In the field of solid cancers, the knowledge about type or subtype-specific mutations is limited. The classification of sarcoma, previously based on the site of the tumor (bone or soft tissue), currently also relies, in selected cases, on mutations associated with specific histological subtypes [19].

Clear cell renal cell carcinoma (ccRCC), which accounts for approximately 75% of cases of renal cell carcinoma (RCC) [20], is characterized in up to 83% of cases by mutations of the Von Hippel-Lindau (VHL) gene [21]. Together with inactivating epigenetic alterations and loss of heterozygosity (LOH), VHL gene mutations contribute to more than 90% of patients exhibiting loss of function (LOF) of the VHL protein (pVHL) [22].

ccRCC is an aggressive form of RCC which typically shows a highly vascularized stroma, haemorrhagic areas [23–25] and frequent intravenous tumor embolization [26], suggesting that CCC may represent interesting prognostic and predictive markers to monitor disease progression and response to therapy.

Therefore, reliable identification of CCC in ccRCC patients, although considered as a difficult task [27], appears to be an interesting liquid biopsy approach.

This study has been planned to compare CRC cytomorphological analysis with their single-cell VHL-targeted genetic analysis. Our results show that all the CCC have been found to carry the same VHL mutation detected in the tumorous tissue. Furthermore, we found that the majority of CRC-UMF also carry the same mutation found in the tumor tissue, suggesting their tumorous nature.

## RESULTS

### Genetic analysis of DNA from tumor tissues and corresponding leukocytes

Tumor tissue DNA analyses from the 30 patients included in this study revealed that four patients (13.3%) had no detectable VHL mutations in their tumor samples (Table 1). At genetic level, 25 of 30 tumor samples (83.3%) were characterized by mutations in the VHL coding sequence. Interestingly, three patients (10%) harboured two simultaneous VHL mutations in their primary tumor sample, each located on a different exon of the VHL gene (Table 2). The rest of the cohort presented single VHL mutations located either on exon one (33.4% of patients), exon two (13.3% of patients) or exon three (30% of patients) of the VHL gene. We identified 18 distinct VHL mutations including nine (50%) mutations located on exon one, four (22%) mutations on exon two and five (28%) mutations on exon three. Genetic analysis of tumor DNA samples revealed that 38.9% of patients had deletions inducing frameshifts, 44.4% presented transversions and 16.7% harboured transitions. All VHL mutations found were investigated to determine their phenotypic impact on pVHL functions by searching four distinct databases (see Methods and Table 1). Additionally, all missense mutations found in our cohort were further investigated by using a polymorphism phenotyping program (PolyPhen). It is important to note that the 85% sensitivity and 44% specificity of PolyPhen predictions for loss-of-function mutations [28] may explain the discrepancies between the reported impact of a missense mutation found in the literature and the PolyPhen prediction obtained for the same mutation (see Table 1).

**Table 1:** Types of VHL mutations detected in ccRCC tumorous tissues

Exon, Codon	Detected in patient n°	Nucleotide change	Type of mutation (codon change)	Amino acid change	Zygosity	Impact on pVHL functions reported in the literature	PolyPhen prediction for missense mutations (score)
Wild Type	15, 18, 22, 25	Not applicable					
1, 9	03	c.27G>T	Transversion (GAC>TAC)	D9Y	Heterozygous	Missense: Location on CpG island suggests impact on transcription initiation (newly identified)	Possibly damaging (0.484)
1, 18	01, 07	c.53C>A	Transversion (GCA>GAA)	A18E	Heterozygous	Missense: possibly pathogenic (binding to unknown target altered) [81]	Benign (0.079) #
1, 61	09	c.183C>G	Transversion (CCC>CCG)	P61P	Heterozygous	Silent mutation: no functional impact [82]	−
1, 65	20	c.194C>A	Transversion (TCG>TAG)	S65X	Heterozygous	Truncation: loss of all functions on one allele [82]	−
1, 69	04	c.205-206delCG	Frameshift (CGC>delCG)	E69fsX62	Homozygous	Truncation: biallelic loss of all functions [82]	−
1, 88	06, 19	c.263G>A	Transition (TGG>TAG)	W88X	Heterozygous	Truncation: loss of all functions on one allele [83]	−
1, 92	12	c.275delA	Frameshift (GAC>delA)	D92fsX67	Homozygous	Truncation: biallelic loss of all functions [82]	−
1, 100	27	c.299delC	Frameshift (ACG>delC)	T100fsX59	Homozygous	Truncation: biallelic loss of all functions [82]	−
1, 109	08, 29	c.327delC	Frameshift (ATC>delC)	H109fsX50	Homozygous	Truncation: biallelic loss of all functions [82]	−
2, 116	01	c.346C>G	Transversion (CTT>GTT)	L116V	Heterozygous	Missense: pathogenic (altered 3D conformation) [84]	Benign (0.235) #
2, 118	05, 10, 16	c.353T>C	Transition (CTC>CCC)	L118P	Heterozygous	Missense: pathogenic (HIF-1/2ɑ accumulation) [84]	Probably damaging (1.000)
2, 140	21	c.418delC	Frameshift (CTC>delC)	L140fsX19	Homozygous	Truncation: biallelic loss of all functions [85]	−
2, 145	03	c.435G>T	Transversion (CAG>CAT)	Q145H	Heterozygous	Missense: pathogenic (HIF-2ɑ accumulation) [86]	Probably damaging (0.988)
3, 158	23	c.472delC	Frameshift (CTG>delC)	L158X	Homozygous	Truncation: biallelic loss of all functions [87]	−
3, 163	02, 14, 17	c.486delC	Frameshift (TGC>delC)	L163fsX7	Homozygous	Truncation: biallelic loss of all functions [87]	−
3, 176	24	c.526A>T	Transversion (AGG>TGG)	R176W	Heterozygous	Missense: no functional impact on protein [88]	Probably damaging (1.000) #
3, 183	04, 11, 26, 28	c.548C>A	Transversion (TCG>TAG)	S183X	Homozygous	Truncation: biallelic loss of all functions [89]	−
3, 207	13	c.620C>T	Transition (GCA>GTA)	A207V	Heterozygous	Missense: pathogenic (unknown mechanism) [90]	Benign (0.000) #

^#^Discordant PolyPhen predictions compared to the reported impact of VHL mutations in the literature.

**Table 2:** VHL genetic profiles detected in CRC and corresponding tumor samples

Patient	VHL mutations found in primary tumor DNA	Number of single CRC	Number of CCC (CRC-MF)		Number of CRC-UMF		
			With same mutations as primary tumor	Without VHL mutation	With same mutations as primary tumor	With different mutations than primary tumor	Without VHL mutation
01 #	c.53C>A c.346C>G	8	5	0	3	0	0
02	c.486delC	10	7	0	3	0	0
03 #	c.27G>T c.435G>T	6	2	0	4	0	0
04^*^ #	c.205-206delCG c.548C>A	5	4	0	1	0	0
05	c.353T>C	13	5	0	5	3	0
06	c.263G>A	17	2	0	13	2	0
07	c.53C>A	9	2	0	7	0	0
08	c.327delC	7	0	0	6	1	0
09	c.183C>G	18	10	0	7	1	0
10	c.353T>C	8	1	0	6	1	0
11	c.548C>A	8	2	0	5	1	0
12^*^	c.275delA	2	0	0	2	0	0
13	c.620C>T	7	3	0	2	2	0
14	c.486delC	1	0	0	1	0	0
15	**Wild Type**	4	0	0	0	0	4
16	c.353T>C	1	0	0	1	0	0
17	c.486delC	8	1	0	7	0	0
18	**Wild Type**	3	0	0	0	0	3
19	c.263G>A	11	3	0	8	0	0
20	c.194C>A	15	3	0	8	4	0
21	c.418delC	6	0	0	4	2	0
22	**Wild Type**	9	0	5	0	0	4
23	c.472delC	2	1	0	1	0	0
24	c.526A>T	2	0	0	2	0	0
25	**Wild Type**	7	0	2	0	0	5
26	c.548C>A	4	3	0	1	0	0
27	c.299delC	6	1	0	3	2	0
28	c.548C>A	5	2	0	1	2	0
29	c.327delC	3	0	0	3	0	0
**TOTAL**		205	57	7	104	21	16

^*^Metastatic patients.

^#^Patients with double mutations in primary tumor and single CRC.

Importantly, all patients exhibited a wild type VHL gene sequence in the DNA from the corresponding single leukocytes tested as reference. Those results demonstrate the accuracy of our molecular data and confirm the absence of germline VHL mutations in our cohort. Overall, 92 individually microdissected leukocytes gave informative genetic data of sufficient quality, providing at least three single-cell controls per patient.

### Cytopathological and molecular analysis of circulating rare cells (CRC)

Among the 30 patients included in this study, 29 had detectable CCC and/or CRC-UMF in their blood samples. From those 29 patients, 327 CRC were individually laser microdissected, their DNA was lysed and preamplified before targeted amplification of the three VHL exons. Among those 327 CRC, we obtained the amplification of the five PCR products covering the three VHL exons and the PCR products from the three control amplifications for 205 single cells, representing an amplification success rate of 62.7%.

Cytopathological reading performed blindly before single-cell laser capture microdissection allowed to classify the CRC as circulating cancer cells (CCC) or CRC-UMF as described in the Methods section. Based on cytopathology, 64 cells derived from 20 patients were classified as CCC and 141 cells from 29 patients as CRC-UMF. No correlation was found between the presence of CCC and/or CRC-UMF and clinicopathological data.

**Figure 1:**
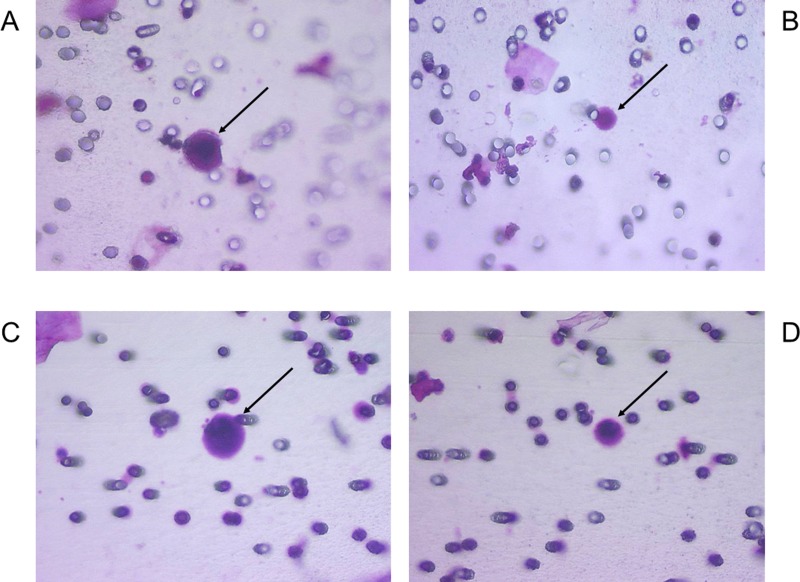
Examples of morphological features of CRC with corresponding VHL alterations (**A**) CCC with c.353T>C mutation; (**B**) CRC-UMF with c.353T>C mutation; (**C**) CCC with c.183C>G mutation; (**D**) CRC-UMF with c.183C>G mutation; with black arrows pointing to each cell of interest.

Our molecular data, obtained blindly, validated the presence of a VHL mutation in 57 of the 64 CRC identified as CCC and in 125 of the 141 CRC identified as CRC-UMF by the cytopathologists. Examples of morphological profiles are shown in Figure 1. The remaining seven CCC and sixteen CRC-UMF without VHL mutation were all found to derive from the four patients harbouring a wild type VHL sequence in their tumor tissue. Our molecular results showed a complete correlation of absence of VHL mutation in the tumor and in the corresponding CCC and CRC-UMF. Yet, those 23 CRC derived from the four patients without VHL mutation in their tumor tissue could not help the diagnosis of CRC using genetic data. Remarkably, the type of VHL mutation detected in the 57 validated CCC, as well as in 104 CRC-UMF, correlated exactly with that detected in the corresponding tumor samples, strongly suggesting the neoplastic nature of at least some cells classified as CRC-UMF by the cytopathologists. We also found 21 CRC-UMF derived from 11 patients which exhibited a distinct VHL mutation from that found in the corresponding tumor tissue, raising the issue of their possible pre-tumor nature and/or origin from a minority tumor cell clone.

One hundred and sixty-one CRC (CCC and CRC-UMF) isolated from the blood of these 25 patients showed mutations of the VHL gene identical to and 21 CRC-UMF displayed mutations of the VHL gene different from those detected in the corresponding tumor tissue. In these 182 CRC detected in 25 patients, we found 18 different VHL mutations affecting the three exons of the VHL gene: seven are missense mutations that induce an amino acid change within the VHL protein in eight patients (26.7%), one is a silent mutation with no effect at protein level found in a single patient, seven are deletions causing frameshifts and three are nonsense mutations that generate premature stop codons and result in a truncated VHL protein for 16 patients (Table 1).

**Figure 2:**
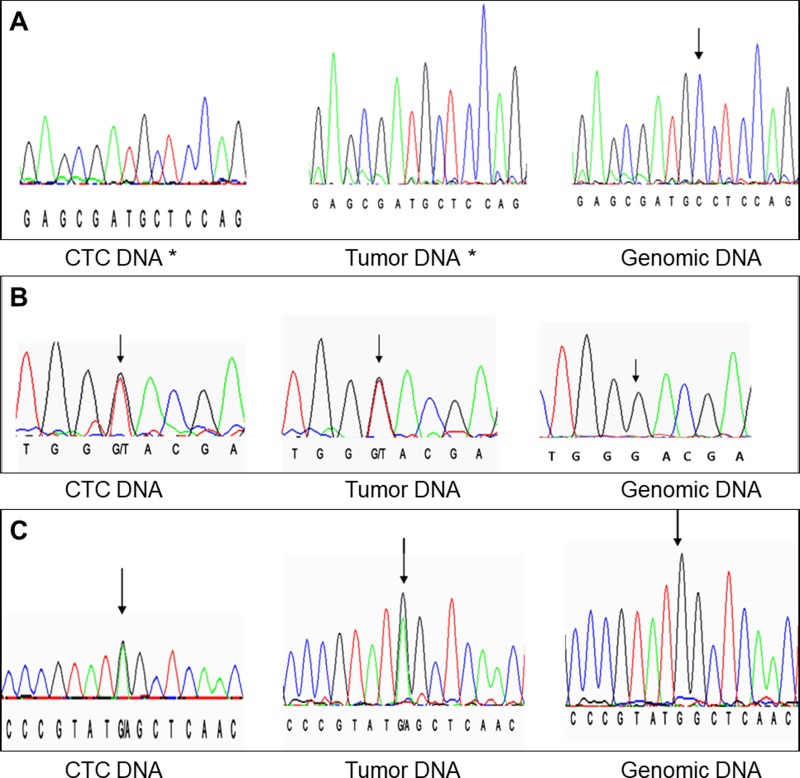
Examples of matching DNA profiles (**A**) exon 3 codon 163 (c.486delC); (**B**) exon 1 codon 9 (c.27G>T); (**C**) exon 1 codon 88 (c.263G>A). Black arrows point to each nucleotide of interest, except when the nucleotide is deleted by the mutation (^*^).

We found an identical VHL gene sequence in 100% of the CCC and corresponding tumor samples from the 20 patients, including two patients harbouring wild type VHL sequences in their CCC and corresponding tumor samples. We also found an identical VHL gene sequence to that of the corresponding tumor samples in 85.1% of CRC-UMF from the 29 cases, including four patients harbouring wild type VHL sequences in all their CRC-UMF and corresponding tumors. Examples of matching molecular profiles are shown in Figure 2 and mutational status of all CRC compared to corresponding tumor samples are detailed in Table 2. Interestingly, double mutations were found in 19 of the 205 CRC analysed (9.3%), all belonging to three patients bearing the same double mutations in their tumor tissues (Table 2).

In single-cell analyses, heterozygous mutations may be missed in case of preferential amplification of one allele, *i.e.* by allele drop out (ADO) [29]. By contrast, ADO is not likely to happen in tumor tissue analyses because genetic analyses are addressed to a large number of tumor cells. Still, we found concordance of genetic profiles identified in all 64 validated CCC and in 120 CRC-UMF, concerning both homozygous and heterozygous VHL mutations, as compared to corresponding tumor samples. We think that this is related to the strict selection of Primer Extension Preamplification (PEP) products using high level quality assessment based on three control PCRs (amplifying reference DNA fragments of different sizes - see Methods) and five PCRs amplifying the three VHL exons which excluded over a third of the PEP-derived DNAs from microdissected single cells. However, our analysis could not detect large deletions across the VHL gene because single-cell PCR performed on fixed and microdissected cells can only amplify short DNA fragments. We used primers designed to cover the intron-exon boundaries of the VHL gene but did not find any splice-site mutations at intron-exon boundaries in our cohort.

Our results show a 100% specificity of the cytopathological diagnosis of CCC, based on concordance of VHL genetic profiles obtained from all the CCC and corresponding tumor samples. Furthermore, our data show that the cytopathological diagnosis of CRC-UMF also matches, in a large proportion of cells (85.1%), with the presence of the same VHL mutation found in the tumor tissue, thereby strongly suggesting their tumor cell nature.

## DISCUSSION

Cancer diagnosis is known to be based on histopathological and/or cytopathological assessment. However, the diagnostic identification of tumor cells circulating in blood is known to be a challenging task [1] due to the morphological heterogeneity of Circulating Rare Cells (CRC) including non-tumorous, non-haematological cells to be distinguished from CCC. Hence, we thought that cytopathological diagnosis in the field of CCC could benefit from complementary molecular analyses.

In this setting, and in order to assess the diagnostic reliability of cytopathology, we have carried out a blinded comparative study, at single-cell level, of cytopathological diagnosis of CCC according to the reference criteria reported by Hofman *et al.* [30, 31] and their genetic analysis. We tested 30 patients with ccRCC, as this particular cancer is characterized by VHL mutations in up to 83% of cases [21], and included, as control samples, the corresponding tumorous tissues, and corresponding individual leukocytes.

CRC-UMF and CCC were identified by cytomorphological analysis in 29 (96.7%) and 20 (66.7%) patients, respectively. However, 25 (83.3%) of the 30 patients harboured a mutated VHL gene in the primary tumor and allowed the comparison based on genetic data. Among them, 18 exhibited 57 CCC (along with 85 CRC-UMF) and 7 exhibited 19 CRC-UMF alone, which were found with the VHL mutational profile also identified in the corresponding tumor tissue. Twenty-one CRC-UMF derived from 11 of these patients were found to bear VHL mutations different from those of the primary tumor. Interestingly, no CCC nor CRC-UMF lacking a VHL mutation were found in the blood of the 25 patients with a VHL-mutated tumor. In contrast, VHL mutations were not found in the 7 CCC nor in the 16 CRC-UMF derived from the four patients with the tumor lacking VHL mutations.

Accordingly, a complete correlation was found between the cytopathological diagnosis of CCC and the detection, performed blindly by their single-cell analysis, of the same VHL sequence found in the tumor tissue, thus validating cytopathological diagnosis

Only few studies have been focused on CTC analysis [25, 32–37] in patients with sporadic ccRCC. Circulating tumor cells derived from ccRCC patients are known to be heterogeneous, prone to EMT and often lack epithelial antigens [38], thereby preventing their capture by epithelial marker-dependent methods [35–37]. In fact, loss of VHL function has been correlated with cellular dedifferentiation and expression of the mesenchymal marker vimentin [39]. In order to capture the maximum number of tumor cells from blood we thus used the ISET^®^ system which is marker-independent and displays an extremely high sensitivity [14].

El-Heliebi *et al.* have also isolated CRC from RCC patients by blood filtration and used immune-molecular analyses to validate CRC cytopathological diagnosis. However, they did not use cancer-specific immunolabelling and molecular tests were addressed to clusters possibly including tumor and non-tumor cells [27]. They concluded that cytopathology alone is not sufficient to allow reliable detection of CTC/CTM but do not provide any diagnostic immune-molecular guideline to identify them.

We found a total of 18 different VHL mutations in our cohort, including 17 which had previously been described. This is not surprising since more than 500 VHL exonic mutations have already been reported in the literature. By interrogating four distinct databases, we determined that only one mutation found in our cohort had not been previously described (Table 1).

VHL mutations can be used as a genetic proof of the cellular tumor nature. In fact they are known to represent the earliest event in ccRCC tumorigenesis [39–45]. Furthermore, in patients diagnosed with ccRCC, several reports have shown that genetic alterations of the VHL locus are the only ubiquitous drivers found in the tumor, including when comparing multiple metastatic sites to the primary tumor [46–49]. Such observations prompted us to use VHL mutations as a marker of tumor nature in patients with ccRCC and a VHL mutated gene in the tumor tissue.

**Figure 3:**
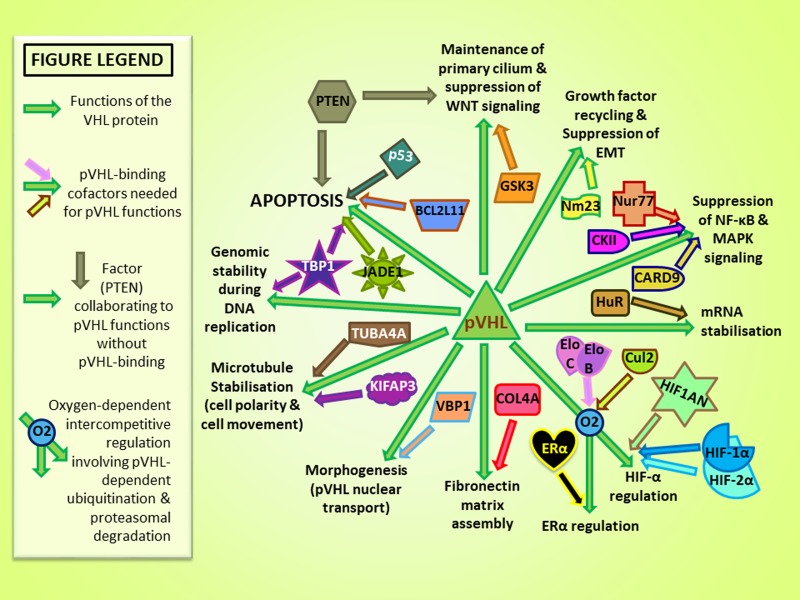
Protein cofactors and major published physiological functions of pVHL [91, 92] Green arrows represent links to pVHL functions and distinctly colored arrows converging with green arrows indicate pVHL-binding cofactors implicated in pVHL functions.

Tumorigenesis is linked to the loss of function (LOF) of the VHL protein (pVHL) (see Figure 3 for pVHL functions). However, although loss of heterozygosity (LOH) at chromosome 3p occurs in over 90% of sporadic ccRCC [50], in a variable proportion of cases [20, 76–78] VHL mutations are not identified in the two alleles. In fact, promoter hypermethylation of VHL with silencing effect, histone modifications affecting transcription and mutations leading to aberrant splicing of mRNA precursors could lead to truncated and/or abnormal VHL protein, thereby contributing to pVHL LOF [54, 55].

In this setting, we stratified our ccRCC patients based on their VHL mutational profiles. As detailed in Table 3, complete loss of functional pVHL due to truncating homozygous VHL mutations was found in 13 patients (43.3%) of our cohort. These mutations, based on the literature [22, 51–53, 56], are a full genetic hallmark of tumor phenotype. In these cases, not only the CCC but also all the CRC-UMF displaying the same VHL mutational profiles found in the tumor tissue are genetically proven to be tumor cells with pVHL LOF. In another group of 10 patients (33%) we found heterozygous VHL mutations in the tumor tissue and in the corresponding CRC (Table 3). In these patients, the pVHL LOF could be related to concomitant epigenetic alterations or undetected monoallelic large deletions. However, two of these 11 patients harboured double missense VHL mutations in the tumor tissue that were found identical in the corresponding CRC (including seven CCC and seven CRC-UMF) and could presumably be located on the two VHL alleles, leading to pVHL LOF.

**Table 3:** VHL-mutations detected in CRC according to their expected functional impact on pVHL

		VHL mutations expected to change pVHL function (*n* = 23)	VHL mutations without expected impact on pVHL function (*n* = 2)	Total number of informative patients (with VHL mutation in the tumor and CRC in blood) (*n* = 25)
**Number of patients**	harboring CCC diagnosed by cytopathology	17	1	18
	harboring CRC-UMF with the same VHL mutation found in the tumor	23	2	25
	harboring CCC or CRC-UMF with the same VHL mutation found in the tumor	23	2	25
**Number of single cells**	classified as CCC by cytopathology	47	10	57
	classified as CRC-UMF & with the same VHL mutation found in the tumor	95	9	104
	classified as CCC or CRC-UMF with the same VHL mutation found in the tumor	142	19	161

We note that our molecular approach did not allow us to explore the full VHL gene sequence. We performed the sequencing analysis of the coding regions of the VHL gene, but could not study the intronic sequences nor the promoter methylation or other mechanisms of pVHL inactivation, because of the limited DNA material which can be obtained from fixed and microdissected single cells. Furthermore, other VHL-neighboring tumor suppressor genes such as PBRM1, BAP1 and SETD2, located on chromosome 3p21, can be targeted by LOH [57] but could not be analysed in the present study.

We also found that 21 CRC-UMF from 11 patients exhibited discordant VHL mutational profiles when compared to corresponding tumor samples. Multifocal ccRCC tumors of distinct origins can be expected in approximately 11.5% of ccRCC cases [58] and could explain the different VHL mutational profiles found in some CRC-UMF with respect to the corresponding tumor sample. However, those CRC could also derive from distinct pretumoral lesions coexisting with the ccRCC [39, 44], thus we considered that the tumor nature of these cells could not be demonstrated.

Our study is the first to combine highly experienced cytopathological analysis according to “reference” criteria [30, 31] with blind single-cell genetic analysis of both CCC and CRC-UMF.

The reliable detection of CCC in the blood of individuals at risk of developing cancer by using ISET^®^ has allowed early detection of lung, breast, renal and ovarian cancers before tumor detection by imaging [13, 59]. The prognostic relevance of CCC detected by ISET^®^ has been demonstrated for patients with melanomas [60] as well as lung [30, 61], colorectal [62], liver [16], pancreatic [63], head and neck [64] and ovarian cancers [65]. Furthermore, the utility of theranostic characterization of CCC detected by ISET^®^ has been demonstrated for non-small-cell lung cancers [66], castration-resistant prostate cancers [67], colorectal cancers [17], hepatocellular carcinomas [68] and melanomas [69].

CCC identification may represent our best chance of “liquid biopsy” for ccRCC patients, in particular because detection of cell-free tumor DNA (ctDNA) in the blood of patients with ccRCC was recently reported as particularly difficult and inefficient [70], as compared to other types of solid tumors [71, 72].

Strikingly, in our study performed blindly, all the 57 CRC defined as CCC by “reference” cytopathological criteria [30, 31] were carrying the same VHL mutation found in the corresponding tumorous tissue, which was always absent in the corresponding leukocytes used as controls. This finding is consistent with a complete specificity of the cytopathological approach following reference criteria. In addition to this, 104 single cells classified as CRC-UMF by morphological examination were also found to carry the same VHL mutation detected in the corresponding tumorous tissue and could be considered as tumor cells. Taking this view into account, in our study, cytopathology appears as having 100% specificity and 72% sensitivity.

In conclusion, our study validates cytopathology performed using reference criteria as a valuable approach to diagnose the presence of cancer cells in blood and provides a proof of principle that single-cell molecular analysis of CRC may complement cytomorphological analysis and improve its sensitivity for CCC detection in patients with ccRCC.

## METHODS

### Patients

Thirty patients with clear cell renal cell carcinoma (ccRCC) scheduled for either partial (*n* = 9) or total (*n* = 21) nephrectomy were enrolled in this study (Table 4). Patients with ccRCC as part of a hereditary VHL disease (bearing germline VHL mutations) were excluded from this study. Our panel consists of twenty-two men and eight women with an average age of 68.5 years (range: 52–78 years). All 30 patients were of the same ethnicity (Caucasian) and recruited at the same hospital (Service d’Urologie, Hôpital Européen Georges Pompidou, Paris, France). Two of our patients presented with metastatic ccRCC. We did not find any unusual clinical characteristics in the four patients without VHL mutations, none of the four was a metastatic patient. Informed consent was obtained from all patients participating in this study. The present study did not include healthy donors. However, 254 healthy donors have been studied in previously published contributions and CCC were never found in their blood [16, 30, 31, 73].

**Table 4:** Patients characteristics

Clinical characteristics	Number of patients (%) (*n* = 30)
**Tumor stage**	
** T1**	17 (56.7 %)
** T2**	2 (6.7 %)
** T3**	7 (23.3 %)
** Tx**	4 (13.3 %)
**Adenopathy**	
** No**	21 (70.0 %)
** N1**	1 (3.3 %)
** N2**	1 (3.3 %)
** Nx**	7 (23.3 %)
**Metastases**	
** M1**	2 (6.7 %)
** M0**	28 (93.3 %)
**Fuhrman nuclear grade**	
** I**	4 (13.3 %)
** II**	13 (43.3 %)
** III**	8 (26.7 %)
** IV**	1 (3.3 %)
** Unknown**	4 (10.4 %)

### Tumor DNA extraction and molecular analysis

Thirty thick sections of frozen tumors were recovered from all thirty patients. These sections were provided by the Department of Anatomy and Cytology of the Necker Hospital. Manual macrodissection of samples was performed after histopathological examination to collect tumor tissue only. Tumor-DNA extraction was then carried out after incubation of the tissue section with Proteinase K (Tris-HCL 50 mmol/L, pH 8, proteinase K 800 g/ml) at 50° C overnight. Proteinase K was then denatured at 94° C for 10 minutes and DNA extraction was performed using the QIAamp^®^ DNA kit (Qiagen, USA) as per manufacturer’s instructions.

Amplification of the VHL gene was performed on extracted tumor DNA by nested PCR using Taq Gold at 0.05 U/μL with 1X PCR Gold buffer (Thermofisher, USA), MgCl2 at 2.5 mM and mixed dNTP at 0.2 mM. All three exons of the VHL gene were independently amplified by applying 2 consecutive cycles of nested PCR in respective total volumes of 40 μl containing 4 μl of extracted tumor DNA and 20 μl containing 2 μl of the first PCR product (PCR1). Primers and conditions used for the amplification of the coding regions of the VHL gene are detailed in Table 5. The first VHL exon being located on a CpG island makes it more difficult to study [74]. We therefore chose to cut this exon in three parts. PCR primers and conditions were optimized to ensure a maximal PCR efficiency and minimal error rate during amplification. Our primers were designed to cover the intron-exon boundaries within the VHL gene; however, they did not cover the non-coding regions of the VHL gene, nor its promoter.

**Table 5:** PCR primers and conditions

Location	Forward primer (5’-3’)	Reverse primer (5’-3’)	Annealing	Amplicon
Exon 1 Part 1	CGCGCGTTCCATCCTCTAC	GGCCTCCATCTCCTCCTCG	55°C	300 bp
Exon 1 Part 2	GAGTACGGCCCTGAAGAAGA	CCGTCGAAGTTGAGCCATAC	Touchdown 65° C to 60° C	215 bp
Exon 1 Part 3	GCCGAGGAGGAGATGGAG	GCTTCAGACCGTGCTATCGT	54° C	248 bp
Exon 2	ACCGGTGTGGCTCTTTAACA	TCCTGTACTTACCACAACAACCTT	56° C	215 bp
Exon 3	GCCACTGAGGATTTGGTTTT	CAAAAGCTGAGATGAAACAGTG	58° C	215 bp

Reactions were incubated in a GeneAmp 9700 thermal cycler (Applied Biosystems, USA) at 95° C for five minutes before 35 cycles of amplification (including 30 seconds at annealing temperature) and a final extension step of five minutes at 72° C were applied. Final PCR (PCR2) products were then purified using the DNA Clean & Concentrator™-5 kit (Zymo Research, USA) as per manufacturer's instructions. Purified PCR2 products were diluted 1:10 in sterile water prior to sequencing of both strands (bidirectional Sanger sequencing) with Big Dye terminators version 3.1 (Applied Biosystems, USA) as per manufacturer's instructions. Sequencing data were collected raw from a 3130xl Genetic Analyser (Applied Biosystems, USA) and analysed using both Sequencing Analysis^®^ and SeqScape^®^ softwares (Applied Biosystems, USA).

All sequences were analysed by visual inspection of the individual sequencing files. Sanger sequencing of DNA extracted from whole populations predictably informs on the dominant clonal population present in each sample. The possible contamination of ccRCC tumor samples with normal infiltrating cells is reported to be 10% or less [75]. Therefore, concerning tumor DNA, heterozygous transitions and transversions were defined as those presenting a single nucleotide change and for which the minor allele peak height represented >20% of the major allele peak. Homozygous mutations inducing frameshifts (insertions/deletions) were defined as those ablating or adding a number of nucleotides not divisible by three and for which the sequence showed a single signal for each nucleotide following the frameshift and no other peaks higher than 20% of the major allele peak over the reading frame of the sequence (at least 20 nucleotides following the frameshift).

### Blood filtration by ISET^®^

Peripheral blood samples (10 ml) were collected on buffered EDTA (EthyleneDiamine Tetraacetic Acid) before surgery, transported to the laboratory within 3 hours after collection and processed by ISET^®^ filtration [14]. Briefly, each 10 mL blood sample was diluted 1:10 with the Rarecells^®^ Buffer (Rarecells Diagnostics, France) containing formaldehyde, incubated with gentle stirring for 10 minutes at room temperature, and filtered on the Rarecells^®^ Device as per manufacturer's instructions. Enriched CRC from each blood sample were recovered fixed on ten circular areas (spots) of the filter.

### Cytopathological staining and diagnostic identification of Circulating Cancer Cells (CCC)

One or more spots (up to ten) of the ISET filter, each one corresponding to 1 ml of filtered blood, were stained with haematoxylin and eosin as described elsewhere [12]. CRC were first analysed and diagnosed by four cytopathologists who confronted their results to come to an agreement, then blindly and individually microdissected for genetic analysis by an independent operator. According to Hofman *et al.* [30, 31], CCC, also called CRC-MF (circulating rare cells with malignant features), are characterized by at least 4 of the following five criteria of malignancy [76]: anisonucleosis (ratio >0.5), nuclei larger than three times the calibrated pore size (i.e. >24 μm), irregular nuclei, presence of tridimensional sheets, and a high nuclear/cytoplasmic ratio. Past studies have also included other criteria such as nuclear hyperchromatism or the size and number of nucleoli [77] which were not considered in this study. CRC exhibiting at least one and up to three morphological features of malignancy were classified as CRC-UMF (circulating rare cells with uncertain malignant features).

### Molecular analysis of CCC/CRC-UMF at single-cell level

Laser microdissection of each individual cell selected was performed using an Eclipse microscope (Nikon, Japan) equipped with the Cell Cut System (Molecular Machines and Industries, Germany). Each microdissected single cell underwent enzymatic lysis and amplification of DNA by the PEP (Primer Extension Preamplification) protocol, as described previously [78]. Amplification of the VHL gene was then performed by nested PCR using the same protocol described above for tumor DNA analysis except that 6 μL of PEP product served as template in the first round of amplification (PCR1) and that PCR2 products were not diluted prior to sequencing. To verify the absence of contamination, an additional tube containing only the lysis buffer (negative control) was inserted at the cell lysis step for each sample tube and run to the end of the PCR assay. We also further added appropriate negative (containing PCR buffer only) and positive (containing 1 ng of genomic DNA) PCR controls.

At single-cell level, PCR analyses may be affected by allele drop out (ADO) due to the loss of signal from one allele [29]. Therefore, we tested the quality of the PEP products by amplifying three distinct genetic targets distributed across the genome. Primers for nested PCR of the three quality control targets: D7S480, D16S539 and D21S1437 have been previously described [79]. We also optimized PCR primers for the amplification of the five fragments covering the three VHL exons and, for further sequencing analyses, selected only cells for which the PEP product could give eight (three controls and five VHL-specific) successful amplifications. These controls and strategy cannot completely ensure the absence of ADO in our results but provide the guarantee that single-cell genetic analyses are performed on PEP-derived DNA of the best quality. It is to be noted that our single-cell studies with PEP preamplification addressed to fixed and microdissected cells have technical limitations which restrict the number of analyses doable per cell to no more than ten (because each nested PCR requires 6 μL of a 60 μL PEP product). Furthermore, the DNA obtained from fixed cells does not enable amplification of large fragments. Consequently, our protocol did not allow us to perform genetic analyses aiming to detect large homozygous or heterozygous deletions across the VHL gene.

We performed bidirectional Sanger sequencing on single cells, as for the tumor tissue samples. Since single-cell analyses, by definition, are not addressed to a mixture of DNA from different cells, we defined heterozygous mutations as those for which the minor allele peak height represented >10% of the major allele peak, as reported elsewhere in the context of fixed single-cell analysis [80]. Similarly, homozygous mutations inducing frameshifts (insertions/deletions) were defined as those ablating or adding a number of nucleotides not divisible by 3 and for which the sequence showed a single signal for each nucleotide following the frameshift and no other signals higher than 10% of the major allele peak for at least 20 nucleotides following the frameshift [80].

### Molecular analysis of genomic DNA from normal leukocytes

Genomic DNA extraction and VHL analyses were performed for each patient on at least three individually microdissected leukocytes using the same protocol as described for single CRC analysis.

### Genotype – phenotype correlations

All VHL mutations found in single CRC and corresponding tumor tissues were investigated to determine their phenotypic impact on pVHL functions by searching the following databases: the VHL Universal Mutation Database (http://www.umd.be/VHL/), the Catalogue Of Somatic Mutations In Cancer (http://cancer.sanger.ac.uk/cosmic), the database of pVHL interactions (http://vhldb.bio.unipd.it), and records of ccRCC in The Cancer Genome Atlas (https://portal.gdc.cancer.gov/). Additionally, all missense mutations found in our cohort were further investigated by using a polymorphism phenotyping program (PolyPhen). PolyPhen uses sequence homology to predict the functional impact of missense mutations based on the degree of conservation of the affected nucleotide throughout evolution. It is important to note that the 85% sensitivity and 44% specificity of PolyPhen predictions for loss-of-function mutations warrant caution when interpreting PolyPhen scores. In fact, 15% of mutations predicted to be benign are functionally damaging and 56% of mutations predicted to be damaging are benign [28]. This may explain the discrepancies between the reported impact of a missense mutation found in the literature and the PolyPhen prediction obtained for the same mutation (see Table 1).

## Abbreviations

ADO: Allele Drop Out
BAP1: BRCA1-Associated Protein
CAIX: Carbonic Anhydrase nine
CCC: Circulating Cancer Cells
ccRCC: clear cell Renal Cell Carcinoma
CGH: Comparative Genomic Hybridization
CRC-MF: Circulating Rare cells with Malignant Features (= CCC)
CRC-UMF: Circulating Rare Cells with Uncertain Malignant Features
CRC-BF: Circulating Rare Cells with Benign Features
CSC: Circulating Stem Cells
CTC: Circulating Tumor Cells
CTM: Circulating Tumor Microemboli
DNA: DeoxyriboNucleic Acid
dNTP: deoxyNucleotide Triphosphates
EDTA: EthyleneDiamine Tetraacetic Acid
EMT: Epithelial-Mesenchymal Transition
HIF: Hypoxia-Inducible Factors (= HIF-1 + HIF-2)
HIF-1α: first alpha subunit identified in HIF
HIF-2α: second alpha subunit identified in HIF
ISET: Isolation by SizE of Tumor/Trophoblastic cells
LOF: Loss Of Function
LOH: Loss Of Heterozygosity
PCR: Polymerase Chain Reaction
PEP: Primer Extension Preamplification
PFS: Progression Free Survival
pVHL: Von Hippel-Lindau protein
RCC: Renal Cell Carcinoma
SETD2: SET Domain-containing protein two
VCAM1: Vascular Cell Adhesion Molecule one
VEGFR: Vascular Endothelial Growth Factor Receptor
VHL: Von Hippel-Lindau gene.
